# Hemobilia after bile duct resection: perforation of pseudoaneurysm into intra-pancreatic remnant bile duct: a case report

**DOI:** 10.1186/s12893-020-00981-8

**Published:** 2020-12-01

**Authors:** Kazuhiro Yoshida, Yuzo Umeda, Masaya Iwamuro, Kazuyuki Matsumoto, Hironari Kato, Mayu Uka, Yusuke Matsui, Ryuichi Yoshida, Takashi Kuise, Kazuya Yasui, Kosei Takagi, Hiroyuki Araki, Takahito Yagi, Toshiyoshi Fujiwara

**Affiliations:** 1 Department of Gastroenterological Surgery and Surgical Oncology, Okayama University Graduate School of Medicine, Dentistry and Pharmaceutical Sciences, Shikata-cho 2-5-1, Kita-ku, Okayama city, Okayama 7000914 Japan; 2 Gastroenterology and Hepatology Department, Okayama University Graduate School of Medicine, Dentistry and Pharmaceutical Sciences, Okayama, Japan; 3 Department of Radiology, Okayama University Graduate School of Medicine, Dentistry and Pharmaceutical Sciences, Okayama, Japan

**Keywords:** Hemobilia, Bile duct resection, Hepatectomy, Endoscopic balloon tamponade, Case report

## Abstract

**Background:**

Hemobilia occurs mainly due to iatrogenic factors such as impairment of the right hepatic or cystic artery, and/or common bile duct in hepatobiliary-pancreatic surgery. However, little or no cases with hemobilia from the intra-pancreatic remnant bile duct after bile duct resection (BDR) has been reported. Here, we report a case of massive hemobilia due to the perforation of psuedoaneurysm of the gastroduodenal artery (GDA) to the intra-pancreatic remnant bile duct after hepatectomy with BDR.

**Case presentation:**

A 68-year-old male underwent extended right hepatectomy with BDR for gallbladder carcinoma. He presented with upper gastrointestinal bleeding 2 months after the initial surgery. Upper endoscopy identified a blood clot from the ampulla of Vater and simultaneous endoscopic balloon tamponade contributed to temporary hemostasis. Abdominal CT and angiography revealed a perforation of the psuedoaneurysm of the GDA to the intra-pancreatic remnant bile duct resulting in massive hemobilia. Subsequent selective embolization of the pseudoaneurysm with micro-coils could achieve complete hemostasis. He survived without any recurrence of cancer and bleeding.

**Conclusion:**

Hemobilia could occur in a patient with BDR due to perforation of the pseudoaneurysm derived from the GDA to the intra-pancreatic remnant bile duct. Endoscopic balloon tamponade was useful for a temporal hemostasis and a subsequent radiologic interventional approach.

## Background

Hemobilia is defined as the extravasation of blood into the biliary tract. It is one of the morbidities related to hepato-biliary-pancreatic surgery, and ranges from minor to severe bleeding, which is life-threatening [1, 2]. The main cause of hemobilia is iatrogenic, such as impairment of the right hepatic or cystic artery, and bile duct [2]. A pseudoaneurysm of the gastroduodenal artery (GDA) is known to cause hemobilia due to common bile duct perforation [3]. However, there has been no reported cases with hemobilia from the intra-pancreatic remnant bile duct after bile duct resection (BDR), to our knowledge. Here, we report a unique case of a 68-year-old man who presented with massive hemobilia due to psuedoaneurysm perforation of the GDA in the intra-pancreatic remnant bile duct after hepatectomy with BDR. Endoscopic balloon tamponade was useful for temporary hemostasis and subsequent radiologic interventional approach.

## Case presentation

A 68-year-old man underwent extended right hepatectomy with BDR and reconstruction for locally advanced gallbladder cancer. Since the primary tumor infiltrated the hepatoduodenal ligament, it was dissected with regional lymph nodes and nerve plexus. Consequently, the proper, right, and left hepatic artery were skeletonized. We did not send para-aortic nodes for frozen section pathology because there was no suspicion of para-aortic node metastasis. Histologic examination revealed well-differentiated adenocarcinoma of the gallbladder at stage IIIB (UICC 7th edition). He was discharged without any complications, such as abdominal abscess, pancreatic fistula, and bile leakage.

**Fig. 1:**
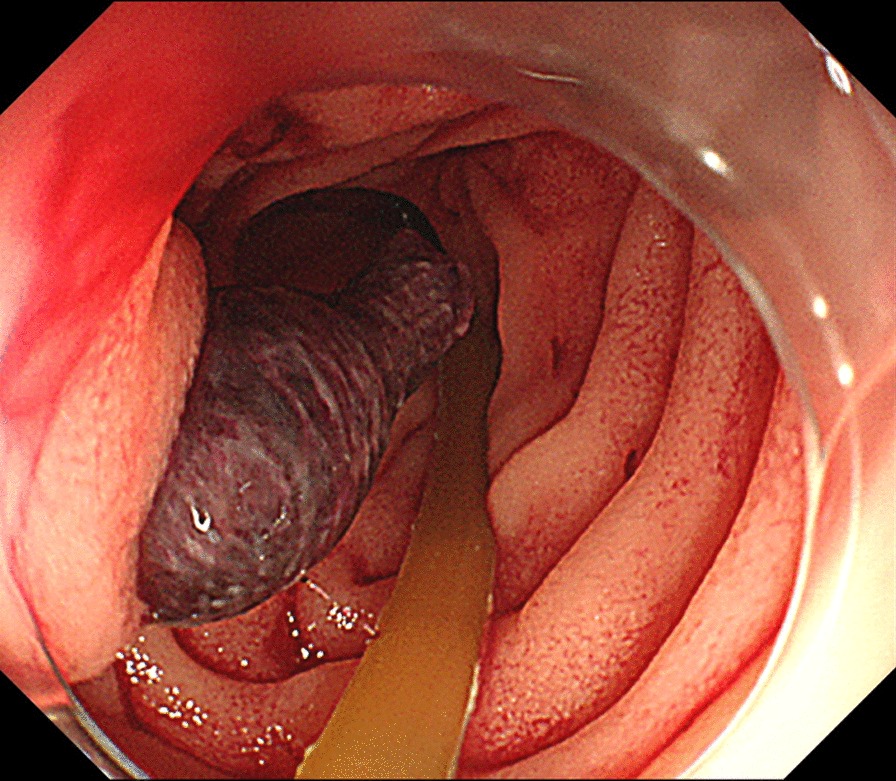
Emergency upper endoscopy shows a blood clot delivered from the ampulla of Vater

**Fig. 2:**
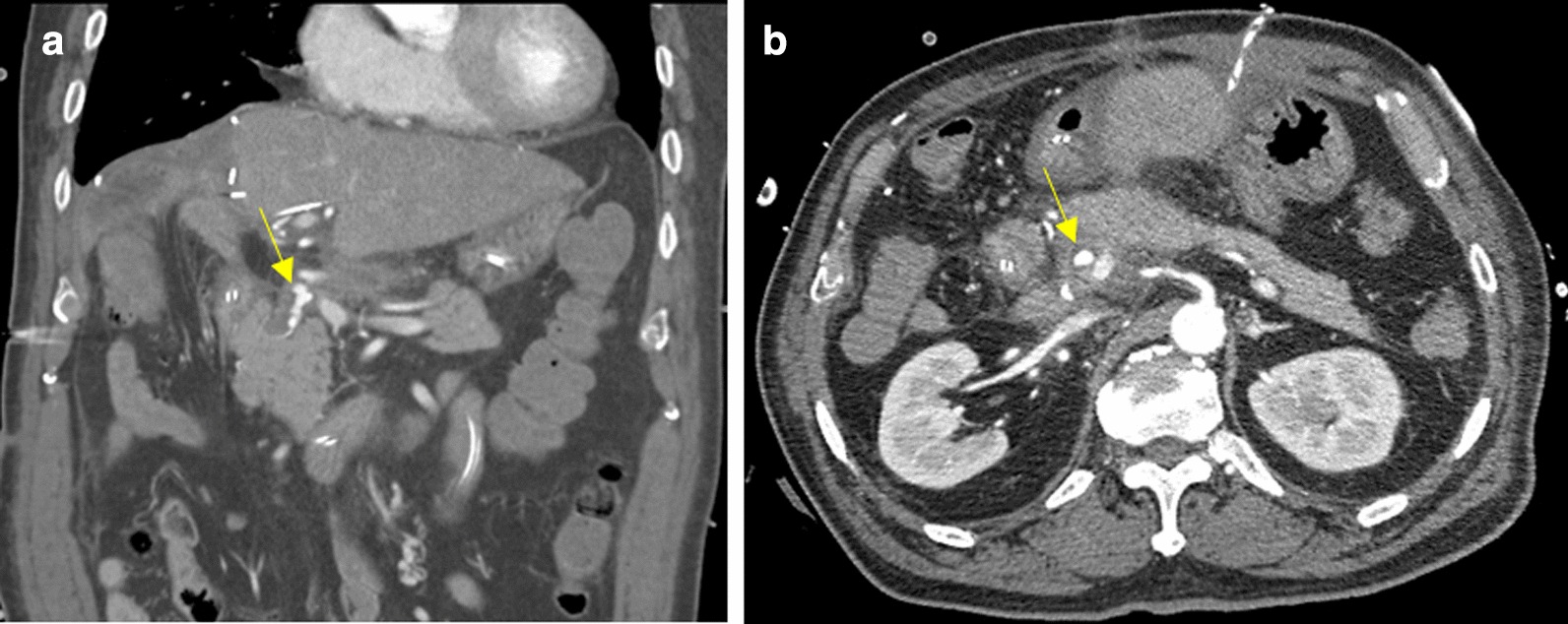
Arrows show the pseudoaneurysm of the GDA in the residual intra-pancreatic bile duct in Coronal (**a**) and Axial (**b**) contrast-enhanced abdominal CT images

**Fig. 3:**
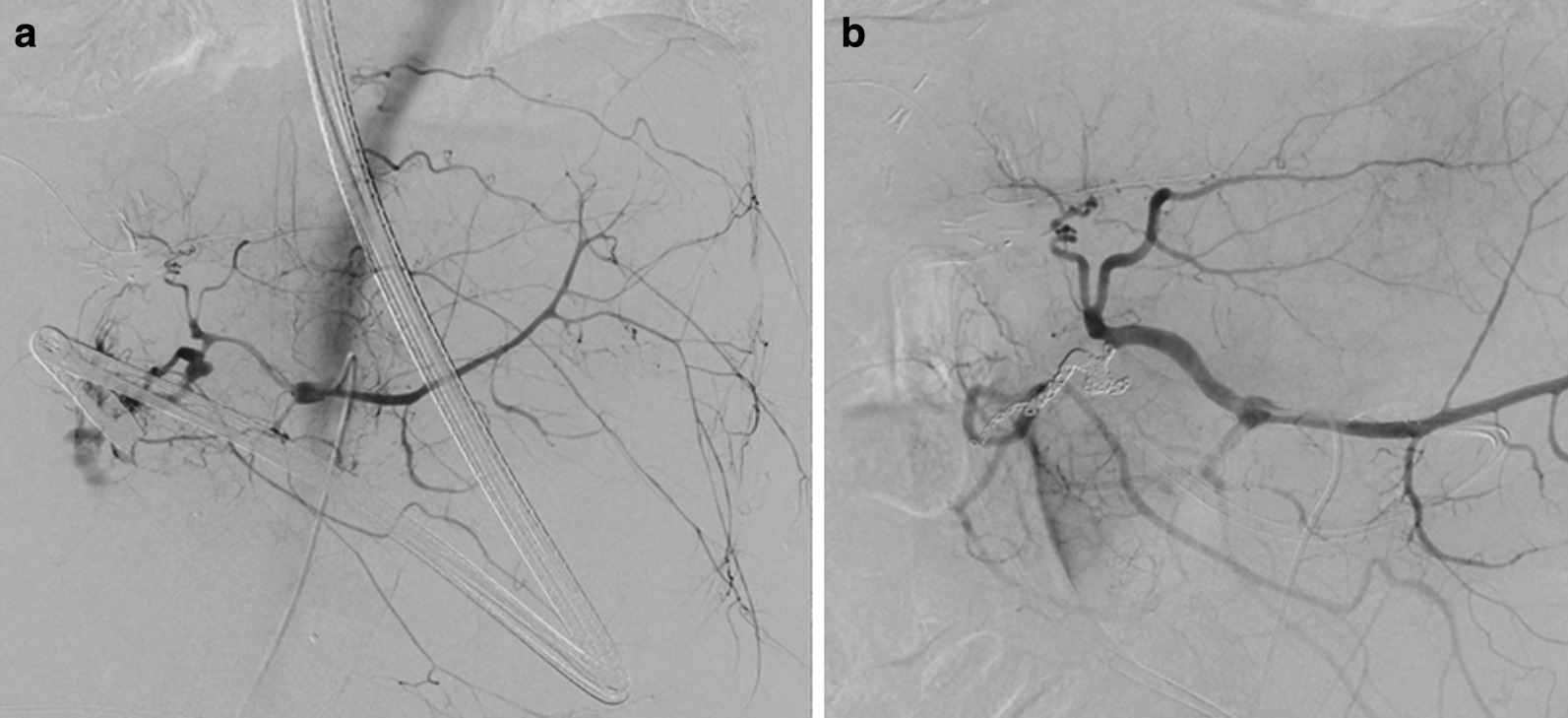
Celiac angiography reveals **a** the rupture of pseudoaneurysm of the GDA into the residual intra-pancreatic bile duct and **b** isolation of the pseudoaneurysm of the GDA with micro-coil

Two months after surgery, he was brought in the Emergency Unit of our hospital for a hematemesis. He was infused rapidly with fluids due to a hemorrhagic shock. An emergency upper endoscopy revealed a blood clot delivered from the ampulla of Vater (Fig. 1). During the examination, the clot was spontaneously blown away, and subsequently massive bleeding occurred. As a result, he entered a hypovolemic shock again with 50 mmHg as systolic blood pressure. Endoscopic tamponade was performed with a balloon catheter that is generally applied to dilate benign esophageal strictures. Fortunately temporary hemostasis was achieved and he proceeded to radiological examination under this temporal hemostatic procedure. Computed tomography and arterial angiography showed that the pseudoaneurysm originated from the GDA and it perforated into the residual intra-pancreatic bile duct (Figs. 2 and 3). After selective distal and proximal embolization of the pseudoaneurysm with micro-coils, massive bleeding was successfully stopped (Fig. 3). He recovered without recurrent bleeding and any other related coil-embolization complications. He is well without any recurrence of cancer and bleeding during the 30-month follow-up.

## Discussion and conclusions

We found two important clinical issues warranting discussion. Hemobilia occurred in the patient with BDR due to a pseudoaneurysm perforation derived from the GDA into the intra-pancreatic remnant bile duct. Endoscopic balloon tamponade was used for temporary hemostasis and subsequent radiological interventional approach.

First, hemobilia occurred due to perforation of the pseudoaneurysm derived from the GDA into the intra-pancreatic remnant bile duct after hepatectomy with BDR. Surgical intervention is known as one of the causes of hemobilia [1, 2]. In most cases, an iatrogenic injury of the right hepatic or cystic artery can induce hemobilia because of the formation of a pseudoaneurysm and rupture to the common bile duct [1, 2]. Few cases described that an unusual position of the GDA (running close to the common bile duct or crossing it higher than usual), can cause hemobilia [3]. In this case, the GDA erodes after radical lymphadenectomy, leading to the formation of a pseudoaneurysm. In addition, it is located anatomically close to the stump of the intra-pancreatic remnant bile duct, which could cause its rupture. To prevent the pseudoaneurysm rupture, isolation of the GDA as well as skeletonized vessels from around tissues by the omentum or the round ligament of liver wrapping may be useful when we perform radical lymphadenectomy.

Second, Endoscopic balloon tamponade was used as temporary hemostasis and subsequent radiological interventional approach. The treatment of hemobilia is achieved by a hemostasis and consequently preventing obstructive jaundice [4]. Hemodynamic parameters were stable with no clear source of bleeding on initial images. Endoscopic retrograde cholangiopancreatography or upper endoscopy is used to detect and manage occult bleeding in a minimally invasive manner. A previous report showed that endoscopic balloon tamponade at the site of a fistula between a peripheral bile duct and a portal vein branch could achieve hemostasis [5], indicating the importance of direct compression of the bleeding point. In this case, a clot in the intra-pancreatic remnant bile duct apparently stopped hemorrhage from the pseudoaneurysm of the GDA. This explained why the patient presented a temporal hemodynamic stability and upper endoscopy was performed eventually. Endoscopic balloon tamponade could temporarily stop arterial hemorrhage because the balloon could compress the ampulla of Vater effectively since it was the lone source.

In conclusion, hemobilia could occur in patients with hepatectomy and BDR due to pseudoaneurysm rupture of the GDA and the residual intra-pancreatic bile duct. Endoscopic balloon tamponade is useful for temporary hemostasis and consequent radiologic interventional approach. The outcome depends on the surgical procedure and the position of the GDA and bile duct should be taken into consideration as a postoperative factor in hepatectomy with BDR. Further studies are recommended to determine the frequency of hemobilia and the hemostatic capacity of endoscopic balloon tamponade because of limitation of these cases.

## Data Availability

Not applicable.

## Abbreviations

GDA: The gastroduodenal artery
BDR: Bile duct resection
